# Strategy‐Level Prodrug Synthesis

**DOI:** 10.1002/chem.202501115

**Published:** 2025-05-24

**Authors:** Paul J. Geaneotes, Paul E. Floreancig

**Affiliations:** ^1^ Department of Chemistry University of Pittsburgh Pittsburgh Pennsylvania 15260 USA

**Keywords:** chemoselectivity, conditional control, functionalization, prodrugs, strategy

## Abstract

Organic synthesis uniquely provides opportunities to access molecules that serve defined purposes. Medicinal chemistry illustrates this attribute well with prodrug design, whereby a drug undergoes a late‐stage conversion to a conditionally responsive active medicinal agent (AMA), being a notable example. Prodrugs are becoming increasingly important in medicinal chemistry but common approaches to introduce biologically responsive groups are limited in the chemoselectivity and scope of available functionalization reactions. This Concept article describes strategy‐level prodrug synthesis, which is a powerful extension of classical prodrug formation that initiates sequences with the objective of introducing functionality early in a sequence to achieve greater scope, site‐selectivity, and chemoselectivity for the incorporation of the biologically responsive group. Examples of functionalization using alkyne hydroamination, Curtius reaction, and alkene metathesis are highlighted along with the use of the prodrugs for biological applications.

## Graphical abstract

Prodrug approaches can be expanded by increasing options for controlling site‐selective functionalization, boosting the range of linking groups, and enhancing the chemoselectivity of reactive group incorporation. This Concept describes strategy‐level prodrug synthesis, whereby these issues are addressed at an early stage of a sequence. New reactions that facilitate this approach and the biological results of the resulting products are highlighted.

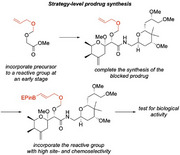

## 1 Introduction

The objective of prodrug synthesis is to optimize the therapeutic benefits of an active medicinal agent (AMA) by masking it as a biologically inactive molecule that is transformed under specific conditions for delivery with maximum efficiency. Masking the AMA can improve absorption, distribution, metabolism, excretion, and toxicity (ADMET) through increased permeability, solubility, and bioavailability.^[^ ^1^ ^]^ The prodrug is typically structured to metamorphose under enzymatic, pH‐dependent, reductive, or oxidative environments. Prodrugs accounted for over 13% of all FDA‐approved small molecule new molecular entities between 2012 and 2022, highlighting the clinical success of applying this strategy.^[^ ^2^ ^]^

The masked AMA contains a moiety (the releasing group) that is intended to cleave upon prodrug exposure to a particular biological agent. The reactive group is often, though not exclusively, connected to the AMA through a linker. The placement of this connection is essential if site‐specific release of the AMA is the objective of the prodrug since it should be inactive until a triggering agent in the desired environment initiates cleavage. The site of attachment is less restrictive if the objective is to improve physical properties. The abundance of enzyme, pH, oxidative, and reductive localization in targeted therapeutic areas has led to the use of carbonyl, phosphate, boronate, acetal, imine, *N*‐oxide, disulfide, thioacetal, and oxime groups as cleavable entities.^[^ ^3^ ^]^ Phosphate, alcohol, amine, carboxyl, sulfamate, amidine, and guanidine groups are common attachment sites^[^ ^4^ ^]^ due to their importance in structure‐activity relationships between AMAs and their biological targets.^[^ ^5^ ^]^

Numerous variations of prodrug design have been developed to respond to a variety of biological conditions. However, FDA‐approved prodrugs heavily rely on only a limited subset of the reported methods. Approximately 94% of FDA‐approved prodrugs rely on enzymatic prodrug cleavage to an AMA, while 5% are pH‐based, and 1% are glutathione‐based.^[^ ^1^ ^]^ Although FDA‐approved drugs are limited in their cleavage mechanisms, they achieve a wide range of specific goals, such as improving bioavailability through increasing permeability, solubility, or tracking (53%), achieving targeted delivery (21%), modulating duration of action (14%), mitigating toxicity (6%), enhancing stability (5%), or promoting synergistic effects (1%).^[^ ^1^ ^]^

**Figure 1:**
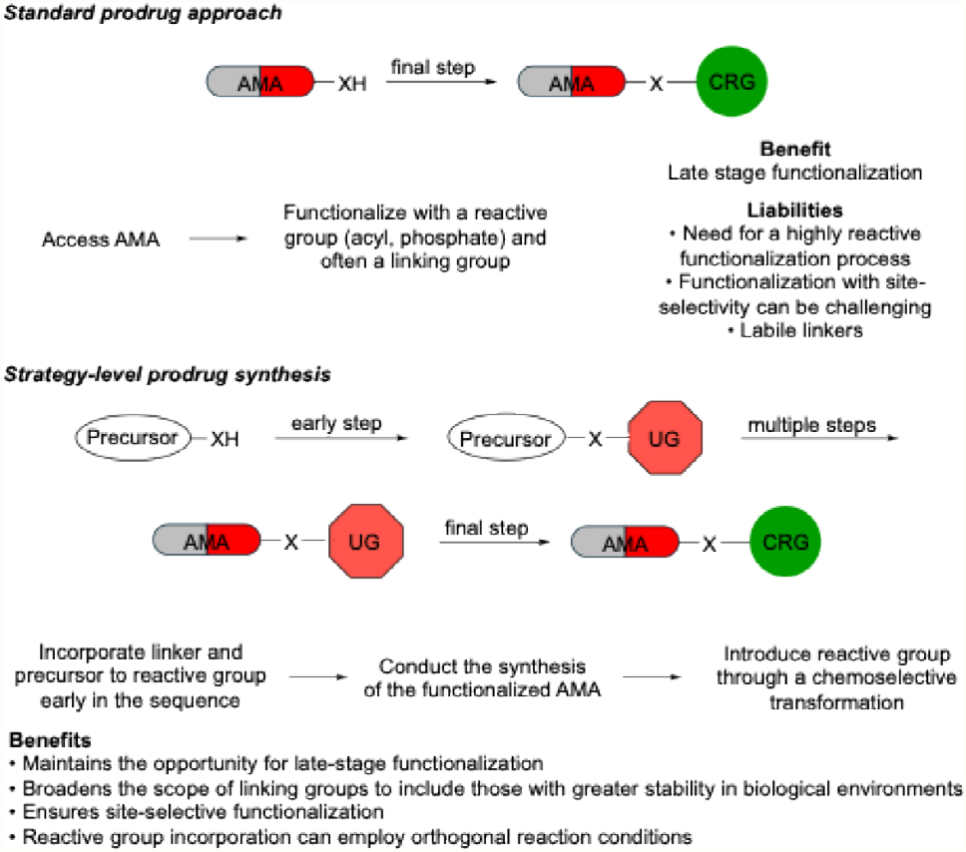
Comparison of standard prodrug synthesis and strategy‐level prodrug synthesis. AMA = Active medicinal agent, CRG = conditionally responsive group, UG = unresponsive group.

The most common approach to prodrug synthesis employs the addition of a releasing group in the final step of a sequence due to the ability of the reactive group.^[^ ^6^ ^]^ This can limit the linkage between the drug and the reactive group to moieties such as esters and phosphates that can be highly electrophilic and, therefore, subject to non‐specific cleavage in biological environments. Expanding the scope of linkers would be desirable for achieving greater stability in biological environments. Late stage functionalizations of complex molecules limit the attachment site to the most reactive group or require protecting group manipulations.^[^ ^7^ ^]^ This can be limiting if the objective of the prodrug is targeted delivery since this will be most effective when the linker is connected to a functional group that is required to elicit a biological response. Designing sequences in which a precursor to the reactive group is added to the requisite moiety at an early stage of the sequence provides an unambiguous approach to site‐selective functionalization. Ideally, this precursor will exhibit unique chemical reactivity that will allow it to be converted to the reactive group in a chemoselective manner. Approaches to prodrug synthesis that utilize the tactic of incorporating a reactive group precursor onto a group that is essential for biological activity through a stable linker at an early stage of a sequence, completing the synthesis of the AMA, then introducing the reactive group through a chemoselective functionalization reaction require the logic of total synthesis rather than derivatization. We propose the term *strategy‐level prodrug synthesis* for this approach (Figure 1). This Concept Article highlights recent advances in reactive group precursor design and selective functionalization that provide guidance on the implementation of this strategy for new innovations in prodrug design.

## 2 Recent Examples of Strategy‐Level Prodrug Synthesis

**Scheme 1:**
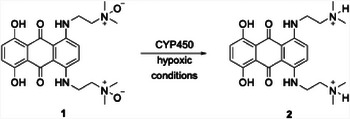
Amine N‐oxide reduction under hypoxic conditions.

An excellent illustration of late stage chemoselective functionalization and conditionally responsive cleavage came from Justin Kim's group at the Dana Farber Cancer Institute.^[^ ^8^ ^]^ This work was inspired by studies that show the reduction of amine *N*‐oxides to amines in cells under hypoxic conditions, as illustrated by the reduction of the prodrug AQ4N (**1**) to its active diamine form **2** (Scheme 1), leading to protonation and DNA intercalation.^[^ ^9^ ^]^ The activation proceeds through the ability of the amine *N*‐oxide to oxidize the reduced form of cytochrome P450 (CYP450) enzymes and, therefore, will be effective exclusively in the absence of O_2_.^[^ ^10^ ^]^ Tumors are commonly hypoxic due to the body's inability to induce sufficient vascularization of the rapidly growing tissue, making hypoxia an attractive trigger for drug release.^[^ ^11^ ^]^

**Scheme 2:**
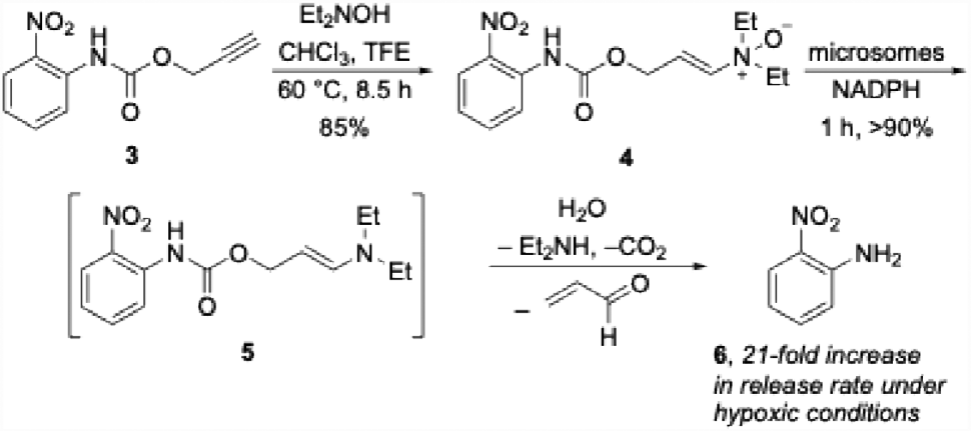
Hypoxic enamine *N*‐oxide reduction and cargo release.

The Kim group hypothesized that enamine *N*‐oxides could be reduced in a hypoxic environment to form enamines, thereby converting an electron‐deficient alkene into an electron‐rich alkene. Incorporating a leaving group at the allylic position results in release upon *N*‐oxide reduction. The pathway is shown in Scheme 2, where the reaction between carbamate **3** with *N,N*‐diethyl hydroxylamine generates enamine *N*‐oxide **4** in high yield and with excellent regiocontrol. Scope investigations showed that regiocontrol in this reaction correlates with the presence of inductively electron‐withdrawing groups at the propargylic position, with phosphates, halides, and acetals promoting excellent selectivity, phenoxy and alkoxy groups showing diminished but useful selectivity, and amino groups showing negligible selectivity. Exposing **4** to microsomes under anaerobic conditions (0% O_2_) promoted reduction to enamine **5** and release of *o*‐nitroaniline (**6**) with, presumably, the generation of acrolein. The release was at least 21 fold faster in the absence of O_2_ compared to studies under normoxic (21% O_2_) conditions. Carbamates and halides were the only groups that were studied for release efficiency.

**Figure 2:**
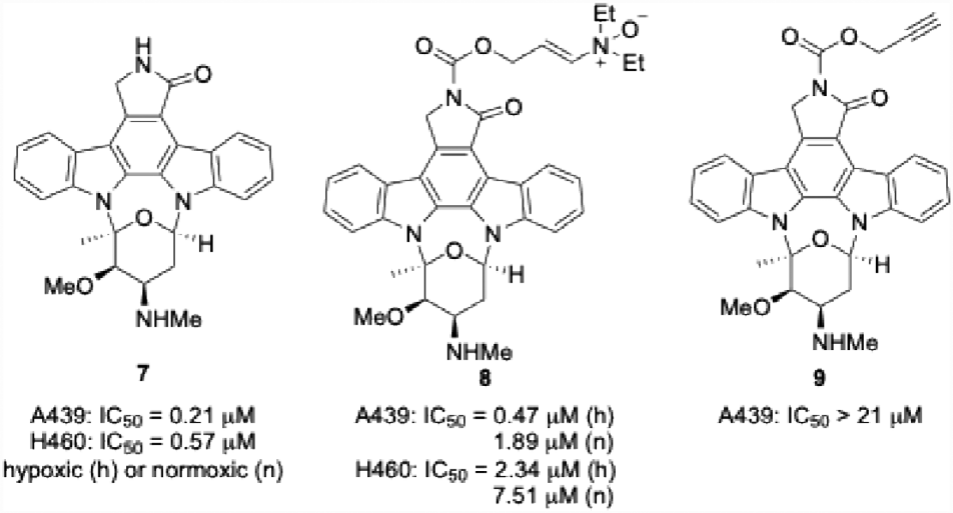
Staurosporine and analogs with cytotoxicity data.

The non‐selective kinase inhibitor staurosporine^[^ ^12^ ^]^ (**7**, Figure 2) served as the basis for testing the capacity of the method to release cytotoxins in cells. The conversion of staurosporine to enamine *N*‐oxide **8** proceeded through a three‐step sequence that also produced the negative control **9**. The authors exposed A431 (epidermoid cancer) and H460 (lung cancer) cells to **7**, **8**, and **9** under hypoxic and normoxic conditions. As expected the response to positive control **7** was insensitive to oxygen content (IC_50_ values of 0.21 µM in A431 cells and 0.57 µM in H460 cells), and the negative control compound **9** failed to generate accurate IC_50_ values, indicating that it is at least 100‐fold less potent than staurosporine. Prodrug **8** proved to be a factor of 4.0 more potent toward A431 cells under hypoxic (0.1% O_2_) conditions relative to normoxic (20% O_2_) conditions (IC_50_ values of 0.47 µM and 1.89 µM, respectively) and 3.2‐fold more potent toward H460 cells (IC_50_ values of 2.34 µM and 7.51 µM, respectively). These results showed that drug release is quite efficient, with the prodrug exhibiting a minimal 2‐ to 4 fold loss of potency compared to the positive control. The authors did not address the difference in potency between the prodrug under normoxic conditions and the negative control, though this result appears to arise from an incomplete suppression of enamine *N*‐oxide reduction in the presence of atmospheric O_2_.

A variation of this method was used to generate an electrophile that can react with nucleophilic residues on proteins. Appending a fluorophore to this electrophile allowed for imaging studies in mice, which revealed that the compound predominantly accumulated in a tumor xenograft. This indicates that hypoxic activation of enamine *N*‐oxides is viable in vivo and, therefore, could prove to be used to deliver drugs in a site‐specific manner to tumors.

This chemistry allows for the late‐stage reactive handle incorporation through a highly chemoselective process. While the precursor to the prodrug was introduced through late‐stage derivatization in these examples, the potential for early introduction in a synthetic sequence is high. This could prove to be valuable for strategy‐level prodrug synthesis. Exciting future directions for this work include expanding the types of functional groups that can be released upon enamine *N*‐oxide reduction and incorporating drug release into the in vivo studies. Additionally, recent work from the Kim group has expanded the scope and regiocontrol of the scope of enamine *N*‐oxide‐forming reactions,^[^ ^13^ ^]^ indicating that the protocol can be used to target a wider variety of systems.

**Scheme 3:**
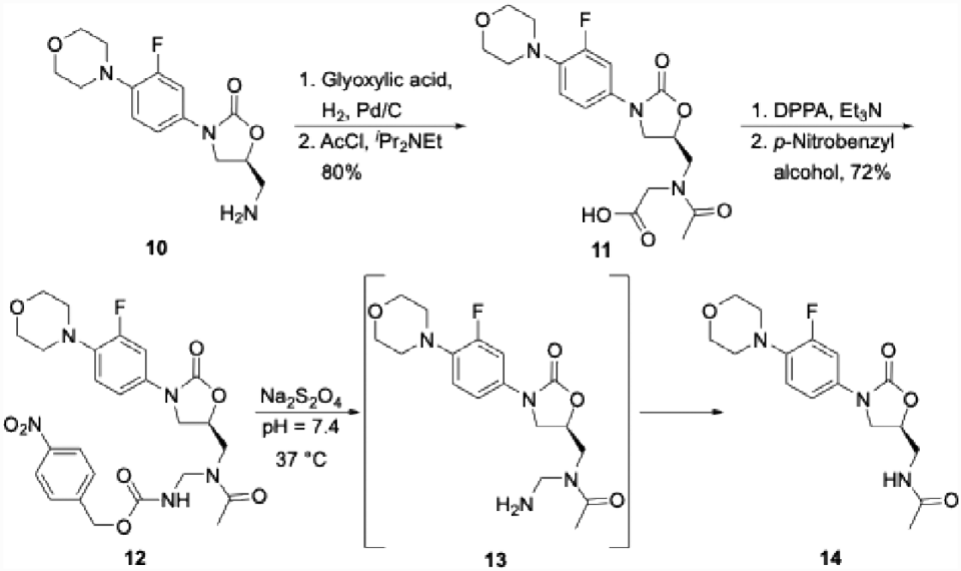
Synthesis and release of an *N*‐acyl aminal‐based prodrug.

David Spring and his group at Cambridge University developed an innovative method for releasing amide groups through a path that employs a Curtius rearrangement.^[^ ^14^ ^]^ Methods for releasing amide groups are still somewhat rare,^[^ ^15^ ^]^ indicating that this work will fill an underserved need. This method proceeds by exposing *N*‐acyl glycine derivatives to diphenyl phosphoryl azide (DPPA) to form an isocyanate that reacts with an alcohol nucleophile and generates a carbamate‐protected aminal species. The alcohol can contain functionality that allows for conditionally controlled amide release. This process is illustrated in Scheme 3. The amine precursor of the antibiotic linezolid^[^ ^16^ ^]^ (**10**) underwent a reductive alkylation with glyoxylic acid followed by acylation to form glycine derivative **11**. Exposing **11** to DPPA and Et_3_N^[^ ^17^ ^]^ followed by heating with *p*‐nitrobenzyl alcohol delivered aminal **12**. Release was demonstrated by treating **12** with sodium dithionite, which rapidly reduced the nitro group. The 1,6‐elimination of the aza‐quinone methide to form aminal **13** and the 1,2‐elimination to release linezolid (**14**) proceeded somewhat more slowly, with the complete release being observed within 24 h.

**Figure 3:**
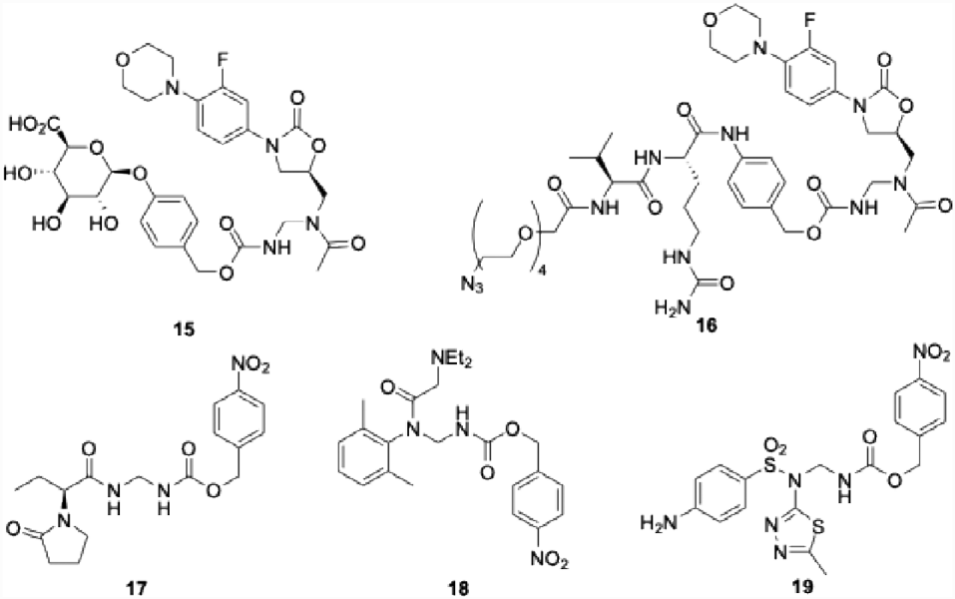
Various amide‐based prodrugs derived from late‐stage Curtius reactions.

The versatility of this method was demonstrated by the preparation of linezolid analogs that release the drug through glycosidase (**15**) and peptidase (**16**) triggers (Figure 3). Additionally, prodrugs that release a primary amide (**17**, releasing the anticonvulsant levitiracetem^[^ ^18^ ^]^), an anilide (**18**, releasing the anesthetic lidocaine^[^ ^19^ ^]^), and a sulfonamide (**19** releasing antibiotic sulfamethizole^[^ ^20^ ^]^) are accessible through the protocol. Exposing **15** to β‐glucuronidase resulted in extremely rapid cleavage and quinone methide release, with complete drug release being observed within 24 h. The described below. Compounds **17**, **18**, and **19** reacted with Na_2_S_2_O_4_ to release their cargo at initial rates that correlate with protease cathepsin B initiated cleavage and release of **16**, with this process being shown in antibacterial studies that will be the nucleofugacity of the drug. While the primary amide and anilide prodrugs reacted only slightly faster than **12**, sulfonamide **19** showed complete release within 4 h. All compounds except for **19** showed excellent stability over a wide pH range and in plasma and did not release their cargo in the absence of the appropriate agent.

Compound **16** was tested for its ability to inhibit *Mycobacterium tuberculosis* growth. This compound, when co‐administered with cathepsin B, showed identical growth inhibition to linezolid in the IC_50_ value and in the magnitude of response after 5 d. No growth inhibition was observed in the absence of cathepsin B or with cathepsin B and a protease inhibitor. This study validated the capacity of the motif to release drugs and effect a biological response in response to an appropriate signal.

This method shows exceptional versatility, with essentially any alkoxy group that can be conditionally cleaved being suitable for incorporation and cleavage. Amide release was the focus of the report, though the method could potentially be expanded to allow for alcohol release from *N,O*‐acetal intermediates. While the derivatizations were conducted at the late stages of the synthetic sequences, the stability of the glycine derivatives that serve as aminal precursors should allow for early incorporation into a sequence to meet the objectives of strategy‐level prodrug synthesis.

**Scheme 4:**
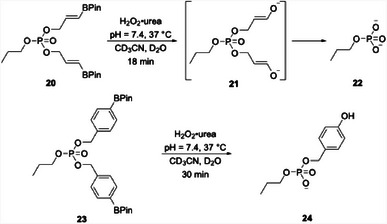
The rate difference in the cleavage of borylated allylic and benzylic phosphates.

Our group at the University of Pittsburgh, in collaboration with Alex Deiters’ group, has been exploring the development and applications of organoboron‐based prodrugs.^[^ ^21^, ^22^, ^23^, ^24^ ^]^ Borylated benzyloxy groups are commonly used in the design of prodrugs that can be cleaved in the presence of hydrogen peroxide,^[^ ^25^ ^]^ which is present in heightened concentrations in disease states such as cancer,^[^ ^26^ ^]^ neurodegeneration,^[^ ^27^ ^]^ arthritis,^[^ ^28^ ^]^ viral infections,^[^ ^29^ ^]^ and diabetes.^[^ ^30^ ^]^ Our initial forays into oxidatively cleavable boron‐containing prodrugs (**20**) showed that the rate of oxidative cleavage of borylated benzylic groups (**23**) was somewhat slow and inefficient.^[^ ^21^ ^]^ This led us to explore the potential of borylated allyloxy (BAO) groups and α‐boryl ethers in prodrug development. These species release their cargo significantly faster and more efficiently than the borylated benzylic counterparts, as seen in the peroxide‐mediated release of phosphates (Scheme 4).^[^ ^23^ ^]^

**Scheme 5:**
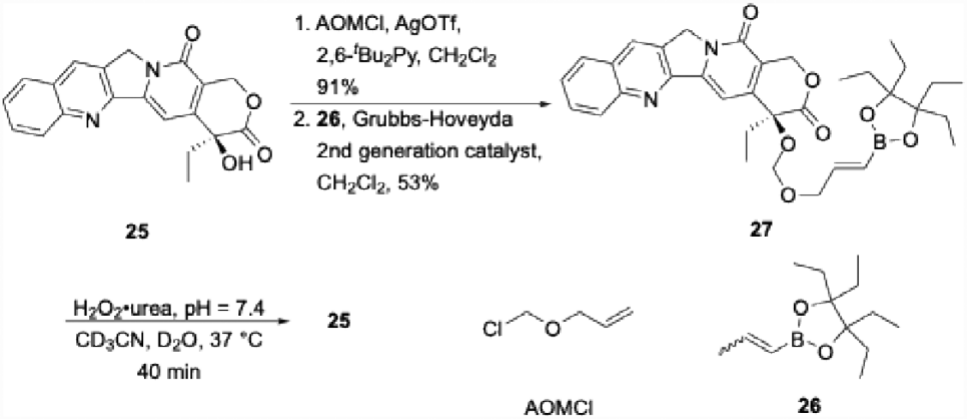
Synthesis and cleavage of an oxidatively labile camptothecin prodrug.

We reasoned that the greater reactivity of the BAO groups could expand the range of functional groups that can be released under oxidative conditions. While borylated benzyloxy groups are generally incorporated into carbonates and carbamates, the BAO groups could potentially release alcohols from ether precursors. The high stability of the ether linkage would create opportunities for incorporating the precursor to the vinyl boronate at an early stage in a synthetic sequence and carrying it through multiple steps. We demonstrated^[^ ^31^ ^]^ that BAO ether formation and cleavage are viable (Scheme 5) through alkylating the topoisomerase inhibitor camptothecin^[^ ^32^ ^]^ (**25**) with allyloxymethyl (AOM) chloride in the presence of AgOTf, followed by a cross‐metathesis with vinyl boronate **26** ^[^ ^33^ ^]^ to form **27**. The use of the ethyl pinacol group^[^ ^34^ ^]^ was advantageous in this chemistry because it provided stability toward the removal of trace ruthenium. Notably, we also showed that vinyl boronates can also be introduced through Cr_2_Zr(Cl)H‐catalyzed alkyne hydroboration^[^ ^35^ ^]^ and through Miyaura borylations^[^ ^36^ ^]^ of alkenyl halides, thereby highlighting the versatility of the design. Exposing **27** to H_2_O_2_•urea at pH = 7.4 and 37 °C resulted in the complete release of camptothecin within 40 min, as determined by monitoring by ^1^H NMR.

**Scheme 6:**
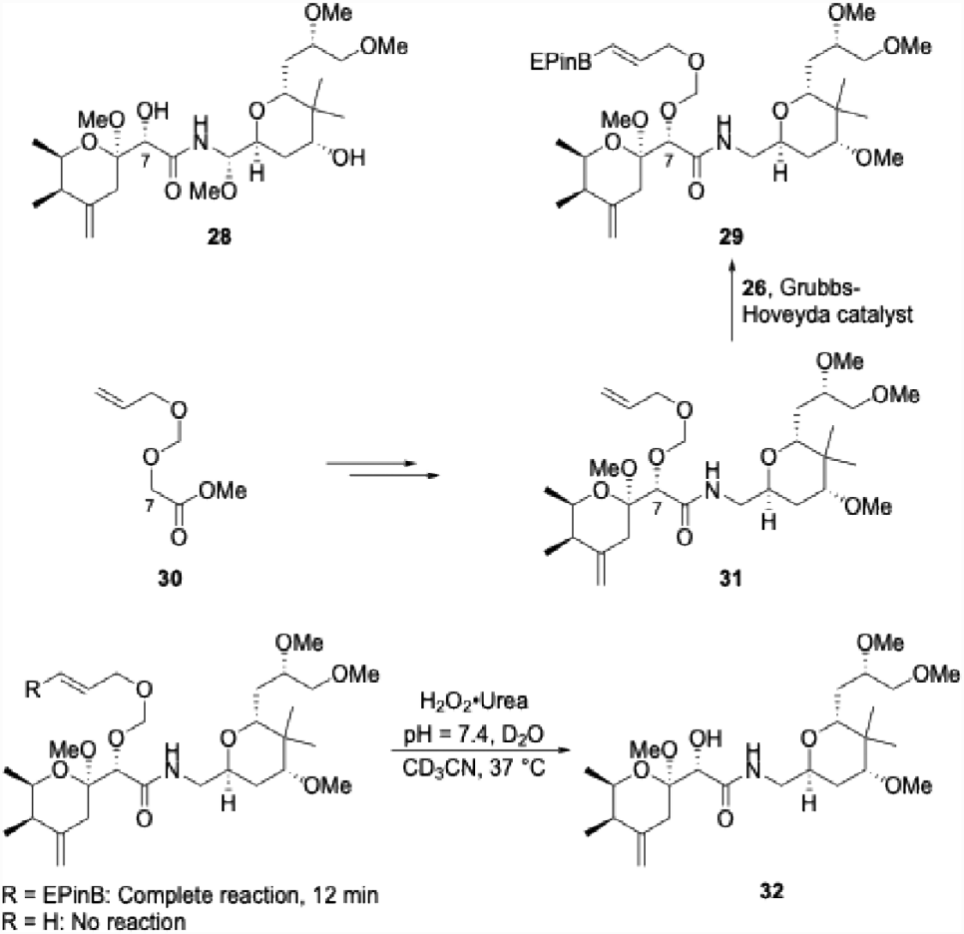
Synthesis and release of a pederin‐based prodrug under oxidative conditions.

Alkenyl boronates are too sensitive to carry through a multistep sequence. However, the AOM group could serve as a viable protecting group that can be converted to an alkenyl boronate as the final synthetic operation. We demonstrated this through the synthesis of a pederin prodrug (Scheme 6). Pederin (**28**) is a natural product derived from beetles that is broadly toxic.^[^ ^37^, ^38^ ^]^ Our prior work^[^ ^39^, ^40^ ^]^ and that of Blunt, Munro, and co‐workers^[^ ^41^ ^]^ on the structure‐activity relationships of this compound led us to propose that compound **29** would serve as a prodrug that would be selectively toxic toward oxidatively stressed cancer cells since the hydroxy group at C7 is essential for biological activity. Glycolate **30** was carried through a variant of our previously reported sequence^[^ ^39^ ^]^ to deliver **31**, which is both the precursor to the prodrug and a negative control for subsequent release and cytotoxicity studies. Cross metathesis of **31** with **26** provided prodrug **29**. Exposing **29** to H_2_O_2_•urea at pH = 7.4 showed an efficient release of **32** while **31**, under the same reaction conditions, was inert.

**Figure 4:**
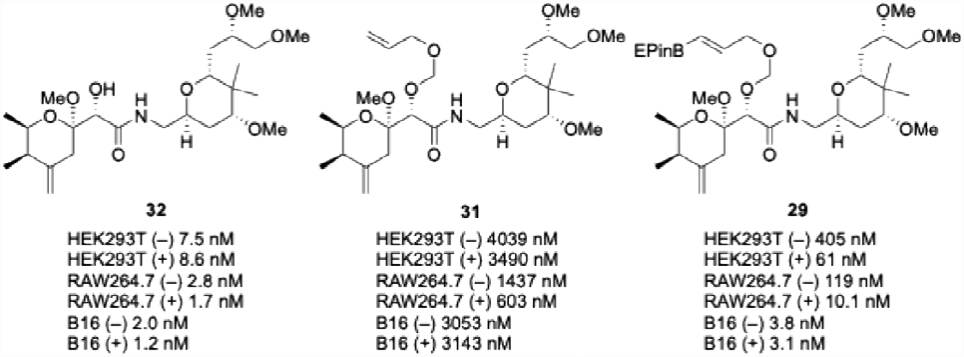
Cytotoxicity data for a pederin‐based prodrug compared to positive and negative controls. (–) Refers to experiments run in the absence of exogenous H_2_O_2_ and (+) refers to experiments conducted in the presence of H_2_O_2_ (100 µM).

Several cancerous and non‐cancerous cell lines were exposed to **32**, **31**, and **29** in the absence and presence of exogenous H_2_O_2_, and select results are shown in Figure 4. Positive control **32** showed potent cytotoxicity toward all cell lines, with IC_50_ values of less than 10 nM. Negative control **31** showed a drop in potency of nearly three orders of magnitude in most cell lines, with only a modest potency boost in the presence of exogenous H_2_O_2_, thereby confirming the essential role of the C7 hydroxy group. Prodrug **29** showed a greater than 50‐fold drop in potency compared to **32** toward HEK293T (epithelial) cells in the absence of H_2_O_2_, with much of the potency being restored upon the addition of exogenous H_2_O_2_. The enhanced potency of **29** compared to **32** in HEK293T cells most likely results from the presence of low concentrations of H_2_O_2_ in most cell lines.^[^ ^42^ ^]^ The potency of **29** against RAW 264.7 (macrophage) cells is higher compared to the HEK293T cells, suggesting a higher level of oxidative stress. Cancer cell lines showed a high sensitivity toward **29**, even in the absence of added H_2_O_2_. The similarity of the responses in the absence and presence of added H_2_O_2_ indicates that the level of oxidative stress in many cancer cell lines is sufficiently high to achieve a full response. The most sensitive cell lines respond to **29** with IC_50_ values that are within a factor of 2 compared to positive control **32**, and with a greater than 100‐fold increase in potency compared to the HEK293T cells, as illustrated by the response to B16 (melanoma) cells. A test compound, which reacts with H_2_O_2_ to release cyclohexanol, formaldehyde, and acrolein, was added to a select group of cell lines in the absence and presence of added H_2_O_2_ and showed no cytotoxicity, even at elevated concentrations. Acrolein is a known toxin when added to cells in a single dose,^[^ ^43^ ^]^ but this work shows that its slow release does not induce a cytotoxic response.

This work fully illustrates the logic of strategy‐level prodrug synthesis, with the precursor to the reactive group serving as a protecting group through a multistep sequence for a group that is essential for biological activity. The ether group creates a stable linkage that inhibits drug release in the absence of endogenous H_2_O_2_ or other agents that are generated under oxidative stress, such as hypochlorite or peroxynitrite,^[^ ^44^ ^]^ that can also initiate cleavage.^[^ ^45^ ^]^ The vast array of transition metal‐catalyzed alkene or alkyne functionalization reactions can be leveraged to expand this strategy beyond oxidatively labile prodrug formation to include groups that can initiate release through a far wider range of conditions.

## 3 Summary and Outlook

Designing molecules that display predictable and beneficial properties is one of the most significant objectives in organic synthesis. Prodrug synthesis is a classic example, whereby an active medicinal agent can be functionalized to improve physical properties or enhance site‐selective drug delivery. This successful strategy shows some limitations, however. Derivatization is either limited to the most reactive group of an AMA or, if the desired function requires derivatization at a different site, protecting groups must be employed. Additionally, the groups that are attached to the AMA are commonly selected for their high reactivity, which can lead to chemical instability in biological environments.

The scope and utility of prodrugs can be greatly enhanced through addressing the limitations described above. Site‐selective functionalization can be addressed by incorporating unique functional groups that can be derivatized through orthogonal chemical transformations that bypass the need for reagents to select between several nucleophilic groups. This strategy will be optimally effective if the precursor to the reactive functional group were introduced at an early stage in the synthesis. This removes ambiguity with respect to site selectivity, allows for the incorporation of a wider array of linking fragments, and provides a handle for chemoselective functionalization. Since the maximally impactful use of this approach involves the logic of total synthesis as applied to *de novo* prodrug generation we have deemed it strategy‐level prodrug synthesis.

The vast array of chemical transformations creates endless opportunities for designing new routes to prepare new prodrug classes. The examples in this manuscript highlight the benefits of utilizing highly chemoselective transformations, including alkyne hydroamination, Curtius reactions, and alkene metatheses, to create new opportunities for drug delivery. These studies provide a roadmap for developing new strategies for prodrug synthesis that will continue to grow in scope as additional methods for introducing conditionally responsive groups into molecules. Crossover strategies are another attractive direction for this approach, whereby linker designs from one method can be combined with the reactive element from another. Moreover, the strategy can be applied to the release of small signaling gases such as SO_2_ and CO, as has recently been demonstrated.^[^ ^46^, ^47^ ^]^ The attributes of developing new and selective agents through *de novo* strategy‐level prodrug synthesis present exciting new directions to expand this important and powerful direction in site‐selective drug delivery.

## Conflict of Interests

The authors declare no conflict of interest.

## Data Availability

Data sharing is not applicable to this article as no new data were created or analyzed in this study.
